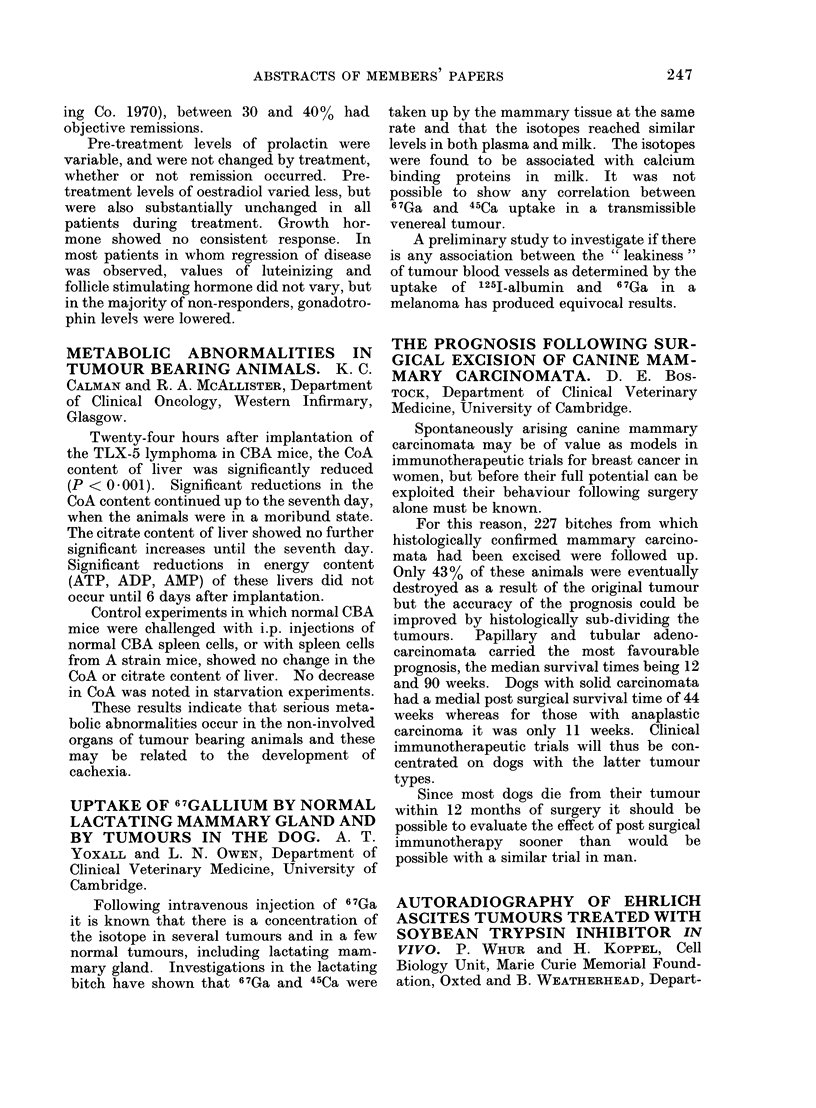# Proceedings: Metabolic abnormalities in tumour bearing animals.

**DOI:** 10.1038/bjc.1975.177

**Published:** 1975-08

**Authors:** K. C. Calman, R. A. McAllister


### METABOLIC ABNORMALITIES IN TUMOUR BEARING ANIMALS.

K. C. Calman and R. A. McAllister, Department of Clinical Oncology, Western Infirmary, Glasgow.

Twenty-four hours after implantation of the TLX-5 lymphoma in CBA mice, the CoA content of liver was significantly reduced ($P < 0{\cdot}001$). Significant reductions in the CoA content continued up to the seventh day, when the animals were in a moribund state. The citrate content of liver showed no further significant increases until the seventh day. Significant reductions in energy content (ATP, ADP, AMP) of these livers did not occur until 6 days after implantation.

Control experiments in which normal CBA mice were challenged with i.p. injections of normal CBA spleen cells, or with spleen cells from A strain mice, showed no change in the CoA or citrate content of liver. No decrease in CoA was noted in starvation experiments.

These results indicate that serious metabolic abnormalities occur in the non-involved organs of tumour bearing animals and these may be related to the development of cachexia.